# Intrinsic Self-Healing Epoxies in Polymer Matrix Composites (PMCs) for Aerospace Applications

**DOI:** 10.3390/polym13020201

**Published:** 2021-01-08

**Authors:** Stefano Paolillo, Ranjita K. Bose, Marianella Hernández Santana, Antonio M. Grande

**Affiliations:** 1 Dipartimento di Scienze e Tecnologie Aerospaziali, Politecnico di Milano, via La Masa, 34, 20156 Milano, Italy; stefano.paolillo@mail.polimi.it; 2 Department of Chemical Engineering, University of Groningen, Nijenborgh 4, 9747 AG Groningen, The Netherlands; r.k.bose@rug.nl; 3 Instituto de Ciencia y Tecnología de Polímeros, ICTP-CSIC, Juan de la Cierva 3, 28006 Madrid, Spain; marherna@ictp.csic.es

**Keywords:** epoxy, composites, self-healing, aerospace, circular economy

## Abstract

This article reviews some of the intrinsic self-healing epoxy materials that have been investigated throughout the course of the last twenty years. Emphasis is placed on those formulations suitable for the design of high-performance composites to be employed in the aerospace field. A brief introduction is given on the advantages of intrinsic self-healing polymers over extrinsic counterparts and of epoxies over other thermosetting systems. After a general description of the testing procedures adopted for the evaluation of the healing efficiency and the required features for a smooth implementation of such materials in the industry, different self-healing mechanisms, arising from either physical or chemical interactions, are detailed. The presented formulations are critically reviewed, comparing major strengths and weaknesses of their healing mechanisms, underlining the inherent structural polymer properties that may affect the healing phenomena. As many self-healing chemistries already provide the fundamental aspects for recyclability and reprocessability of thermosets, which have been historically thought as a critical issue, perspective trends of a circular economy for self-healing polymers are discussed along with their possible advances and challenges. This may open up the opportunity for a totally reconfigured landscape in composite manufacturing, with the net benefits of overall cost reduction and less waste. Some general drawbacks are also laid out along with some potential countermeasures to overcome or limit their impact. Finally, present and future applications in the aviation and space fields are portrayed.

## 1. Introduction

Polymer matrix composites (PMCs) have been broadly studied and employed for several decades now and find an application in different engineering fields, including aerospace. The combination of great load-bearing properties of the reinforcing phase and the versatility of polymers as the matrix element provides advanced lightweight alternatives to heavier materials [1], particularly in the highly demanding aerospace industry, where the application of composites mainly translates into weight reduction, allowing to reduce costs, for example by using less fuel [2]. Fiber reinforced polymers (FRPs) are increasingly used in large passenger aircraft. FRP composites have emerged as a new range of materials, due to their ability to offer substantial advantages over traditional metallic materials in terms of density and fatigue properties. Particularly, the aeronautic industry has found the increased use of thermoset composites in aircrafts, especially in airliners, because of the reduced weight compared to equivalent metal structures. Currently, FRP composites have taken up a major part of the structural mass of some civil aircraft, like the Boeing 787 and Airbus A350 XWB. In the space sector, composites play a crucial role in the design and manufacture of launchers, satellites, spacecraft and instruments, as well as in space habitats such as the International Space Station.

**Figure 1:**
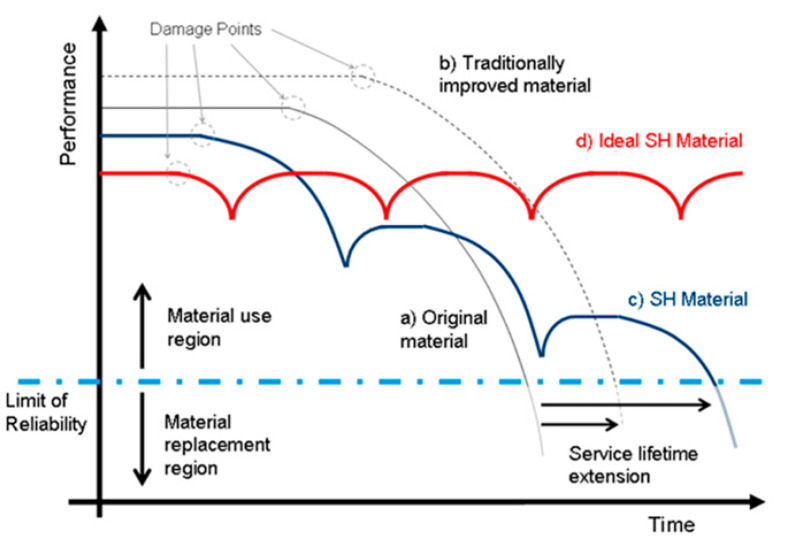
Lifetime extension of engineered materials by implementation of self-healing principles. (reproduced from [5] with permission from Elsevier).

Although PMCs, and especially FRPs, the most used composites in the aerospace sector, offering unique possibilities in terms of strength-to-weight ratio, the inability to plastically deform, thus absorbing energy only via damage creation leading to failure mechanisms like delamination, fiber-matrix debonding, and fiber fracture, clearly curb their use [3]. In addition, poor performances under out-of-plane (i.e., impact) loading are critical. For these reasons, FRPs must be frequently monitored and inspected during service, and since maintenance and repair operations are costly and time consuming, it has been necessary to move from a “damage prevention” approach to a “damage management” perspective, based on a restoring response to the formation of a damage [4]. In this view, the implementation of the self-healing principle to aerospace composite can be beneficial to increase both reliability and safety and it will considerably reduce maintenance costs. From such considerations, the research has shifted towards the design of self-healing materials, which possess the ability to recover a functionality in order to extend their service lifetime [5], as shown in Figure 1. For a general overview on self-healing polymers and their classification, we refer the reader to some recently published comprehensive reviews and inspiring research in the field [6,7,8,9].

Focusing on aerospace, the most widespread polymers used as matrices in FRPs are epoxies. Better characteristics with respect to other thermosetting polymers (e.g., polyesters) in terms of mechanical properties, adhesion to substrates and fibers, resistance to moisture absorption and to corrosive environments make epoxies remarkably suitable for aerospace applications. An additional advantage is their good performance at elevated temperatures owing to high glass transition temperatures, T_g_. Indeed, T_g_ is a pivotal factor to be considered during the design of ad hoc materials for aviation and space industries, as those should not experience softening transitions within the operating temperature range, which are roughly between −50 °C and 60 °C for aeronautical purposes and between −150 °C and 150 °C for space environment [10]. Therefore, T_g_ is expected to exceed these upper temperature limits.

For the design of aerospace epoxy resins, cure temperature usually falls in the 120–135 °C range, but it may increase even up to 180 °C for obtaining high-T_g_ matrices with enhanced resistance to thermal degradation. FRP curing is usually performed in an autoclave or via a closed cavity tool at pressures up to 8 bar, occasionally with a post-cure treatment at a higher temperature. However, lack of toxic emissions (especially when compared with styrene-containing polymer formulations) during the curing process makes epoxy resins much more versatile from a manufacturing standpoint, as open-mold processing (e.g., vacuum bagging or automated lay-up) is possible. Compatible with most composite manufacturing processes, epoxies require low viscosity.

Production techniques for novel self-healing materials to be embedded in composite structures should not deviate from those already employed, otherwise a fast and efficient implementation in manufacturing cannot be accomplished. Furthermore, the added self-healing functionalities should not be achieved at the expenses of other epoxy characteristics: in other words, mechanical and thermal properties of self-healing epoxies have to be comparable to conventional resins.

Although the adoption of self-healing polymeric matrices is forecast to positively affect the composite landscape (in particular for high-end applications), the costs of raw materials and tailored chemistries are generally higher than those currently in use. Therefore, great focus from both a production standpoint and a waste management perspective are desirable and indeed should be addressed as early as possible, taking into account their environmental impact. In this sense, composite recycling has gained the attention of many industries, mainly from automotive and aerospace sectors responsible for more than half of total composites manufacturing. This is not only due to ecology and sustainability-driven strategies, but also to the economic savings for recycling rather than ex novo production. In the context of self-healing polymers, the ability of some healing mechanisms to rearrange the polymer network already provides the basis for reprocessing and recycling, potentially allowing the recovery of thermoset polymers such as epoxies, which has never been achieved until recent years. Many research groups have addressed the issue, providing encouraging solutions for recovering either the matrix material or the reinforcement, and in some instances even both at the same time, though with some drawbacks.

Many reviews, communications and book chapters on the state-of-the-art of self-healing and healable polymers, PMCs and FRPCs have been published [1,3,4,6,11,12,13,14,15,16,17,18,19,20,21,22,23,24,25,26,27,28,29,30,31,32,33,34,35,36,37,38,39,40,41,42,43], and some of them are indeed recent [36,37,38,39,40,41,42,43]. This review focuses exclusively on the description of the main intrinsic self-healing epoxy systems found in the literature, both as a standalone material and as matrices in PMCs, with an emphasis on those systems that are particularly suitable for aerospace applications. Moreover, a perspective view of these self-healing materials incorporated into a circular economy model is also highlighted, allowing their partial recovery and/or reprocessing.

## 2. Healing Efficiency

Before delving into the different modes for epoxies to gain intrinsic self-healing features, it is necessary to clarify how healing can be evaluated in a quantitative fashion so that comparative studies between self-healing systems could be carried out. The so-called “healing efficiency”, denoted by *η*, is defined as the rate of recovery of a virgin material property. In most cases, healing assessment requires a controlled and measurable experiment that generates new surfaces due to damage initiation and propagation. After the occurrence of the healing process, the exact same experiment is repeated, so that the outputs of the two tests can be compared. Commonly used material properties that have been used to quantify healing efficiency along with the corresponding equations are listed in Table 1. Which healing efficiency definition is chosen depends on several factors, such as polymer properties, failure mode, self-healing mechanism and sample geometry, among others. As a rule of thumb, recovery of impact damage, compression after impact (CAI) or flexural after impact strength and fracture mechanics testing are more appropriate for evaluating FRPs, as impact damages and delamination are critical factors [44].

Healing efficiency provides a single, very useful parameter to evaluate self-healing performances. However, a few considerations have to be made not to let oneself be misguided by it: (i) as already stated, the added self-healing functionality should not come at the expense of significantly reducing the virgin properties of the material, and because increasing healing efficiency can be achieved not only by increasing the healed material property, but also by reducing the virgin functionality, in all cases the virgin properties and how they change with added self-healing features should be reported and subsequently taken into account when assessing *η* [10]; (ii) with fracture-based definitions of healing efficiency (i.e., fracture toughness, K_IC_, and fracture energy, G_IC_) it is possible to obtain efficiencies greater than 100%, because fracture tests require the presence of a pre-crack in order to perform the measurements, thus potentially underestimating the fracture properties of pristine, un-cracked samples; (iii) sometimes materials do not reach a complete cured state before testing, thus a portion of the recovered functionality may be given by residual curing phenomena, sparked by the healing cycle. Nevertheless, this effect is easily measurable via FTIR or calorimetric analyses, and thereby included in the healing performances assessment. Therefore, it should be noted that it can be inaccurate to directly compare healing efficiencies reported in different publications measured using different functionalities and test protocols.

## 3. Intrinsic Self-Healing Mechanisms

### 3.1. Physical Interactions

**Figure 2:**
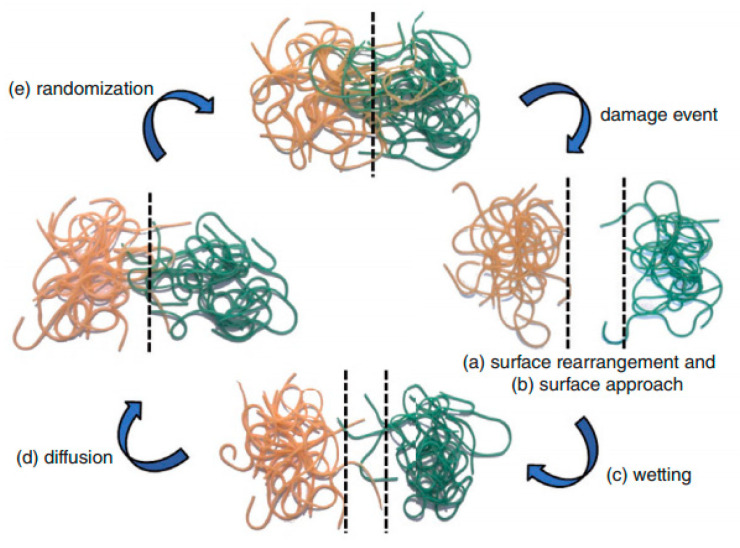
Stages of self-healing mechanism surface rearrangement (**a**), surface approach (**b**), wetting (**c**), diffusion (**d**) and randomization (**e**) (reproduced from [9] with permission from John Wiley and Sons).

Self-healing principles based on physical interactions have been one of the earliest observed self-healing behaviors of engineered polymers. To describe the complex strength recovery process at broken polymer/polymer interfaces, Wool and O’Connor proposed a five-stage mechanism [45], as illustrated in Figure 2. Surface rearrangement (a) and surface approach (b) are the most crucial steps because healing can only take place if the ruptured interfaces are in contact with each other. The wetting stage (c) enables diffusion (d), which results in the entanglement of polymer chains and, therefore, in the subsequent recovery of the mechanical properties of the healed material. Finally, during the randomization stage (e), the complete loss of initial crack interfaces can be observed.

The first mending phenomenon in neat epoxy was reported in 1969 by Outwater and Gerry [46]: fracture energy resulting in local plastic deformation of the areas adjacent to the fracture surface was recovered by heating above 120 °C, enabling healing. Forty years later, Rahmathullah and Palmese further investigated the crack healing behavior of unmodified epoxy-amine systems at different stoichiometric ratios [47]: at stoichiometry, after applying pressure and heating above T_g_, healing occurred due to permanent mechanical interlocking upon cooling below T_g_, resulting in a healing efficiency of about 70%. A combination of physical and chemical phenomena occurs when the formulation is in excess of unreacted epoxy groups: a second mechanism of covalent bond formation takes place due to polyetherification or homopolymerization reactions of unreacted epoxy groups at a crack interface. In this case, the healing efficiency reached values even greater than 100%.

Another series of self-healing mechanisms involving physical interactions can be found in polymer–polymer blends, both miscible and immiscible as described below.

#### 3.1.1. Miscible Polymer Blends

**Figure 3:**
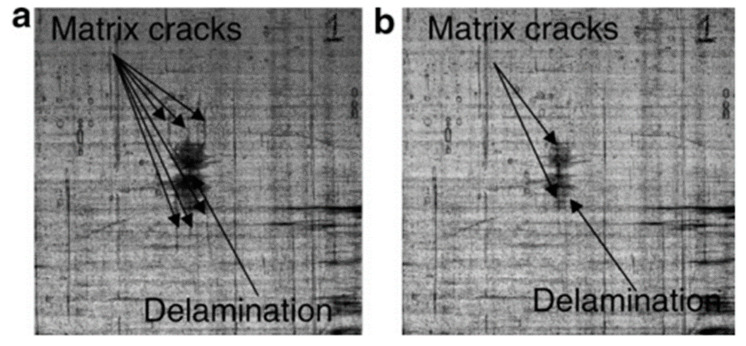
Glass-fiber composite panel impacted: (**a**) before and (**b**) after a healing cycle. (Reproduced from [48] with permission from Elsevier).

Hayes et al. developed a system in which a thermoplastic healing agent is dissolved in epoxy [48]. Upon heating a fractured compact tension (CT) specimen, the thermoplastic material diffuses through the thermosetting matrix, closing cracks and facilitating self-repair. Healing efficiency turned out to increase either with increasing healing temperature or amount of thermoplastic agent. Epoxy systems containing 20 wt% of healing agent showed a 64% recovery of impact strength when healed at 140 °C, while after a 130 °C, 1 h healing cycle, cross ply glass fiber composites whose matrix contained 10 wt% of thermoplastic agent, exhibited more than 30% reduction in damaged area, as assessed via computer-based image analysis (Figure 3). However, since the addition of great amounts of thermoplastic healing agent is detrimental to thermomechanical properties, the system was optimized with 7.5 wt% of healing agent, yielding 43–50% healing efficiency for the resin as a standalone, but confirming 30% recovery of damaged area on cross ply fiberglass composites [49].

Phase separation of an initially miscible blend has been demonstrated by Mather and coworkers [50] with an epoxy/polycaprolactone (PCL) system where a “bricks and mortar” morphology manifests itself after polymerization-induced phase separation of the healing agent: “bricks” and “mortar” refer to the interconnected epoxy spheres and the continuous PCL matrix, respectively. The phenomenon behind healing is called “differential expansive bleeding”: heating to a temperature between the melting temperature (Tm) of PCL (55 °C) and T_g_ of the epoxy (203 °C) resulted in spontaneous surface wetting of samples by molten PCL, as a consequence of differential thermal expansion between the two phases (PCL phase expanding at least 10% more than the epoxy phase), and in the recrystallization of PCL upon cooling, leading to crack closure. An optimal 15.5 wt% PCL provided the best balance between mending performance and mechanical properties, as recovery of peak load was greater than 100% when SENB specimens had been thermally mended at 190 °C for 8 min, under 18.7 kPa compressive stress. Furthermore, this system has been studied to function as a rigid adhesive with excellent reversibility of adhesion via heating above Tm of PCL.

More recently, Michaud and Cohades have extended the analysis of epoxy/PCL systems over a wider range of thermoplastic contents in order to establish a reference epoxy formulation and cure conditions leading to an optimum combination of toughness and stiffness [51,52,53]. Efficient healing is contingent on the presence of a continuous PCL phase able to flow into cracks: this is obtained with concentrations greater than 13.1 vol% PCL, but at the expense of strength and toughness, whose decreases were respectively four-fold and two-fold compared to pure epoxy. However, after a 150 °C, 30 min healing cycle, toughness recovery reached 70–80% efficiency for blends containing 25 and 26 vol% PCL.

Other researchers have studied the same epoxy/PCL system including some embedded reinforcement. As an example, Wang et al. explored the impact of adding carbon nanofibers (CNFs) and showed that a recovery of 78% in bending peak load was achieved with 0.2 wt% CNFs and 10 wt% PCL [54,55]. While the incorporation of E-glass fibers into blends with 25 vol% and 37 vol% PCL via VARIM led to similar morphological developments with the same curing procedures and improved healing efficiencies for higher PCL contents, as well as for a higher number of healing cycles [52]. Furthermore, damage recovery after low-velocity impact has been assessed by means of C-scans through recovery in damage area as well as CAI strength recovery: the system can fully heal damages on the order of 8 J, which correspond to the main concern of maintenance activities in the composite industry (e.g., tool dropped from 1 m height) [53].

#### 3.1.2. Immiscible Polymer Blends

First studies on immiscible healing agents embedded in a composite matrix date back to 1999: Zako and Takano incorporated epoxy particles into a glass fiber-reinforced epoxy composite [56]. Their system included a cold setting type epoxy used as matrix, continuous unidirectionally arranged glass fibers as reinforcement, and a particle-type thermosetting epoxy adhesive, which worked as an actuator to repair damage upon melting. The capability of repairing damages assessed by static bending and fatigue tests on SENT specimens was proven in terms of almost full stiffness recovery and extended residual fatigue life. However, the definition of immiscibility here can be questionable, since the healed material became actually a single component system, while the virgin material is a two-phase system.

**Figure 4:**
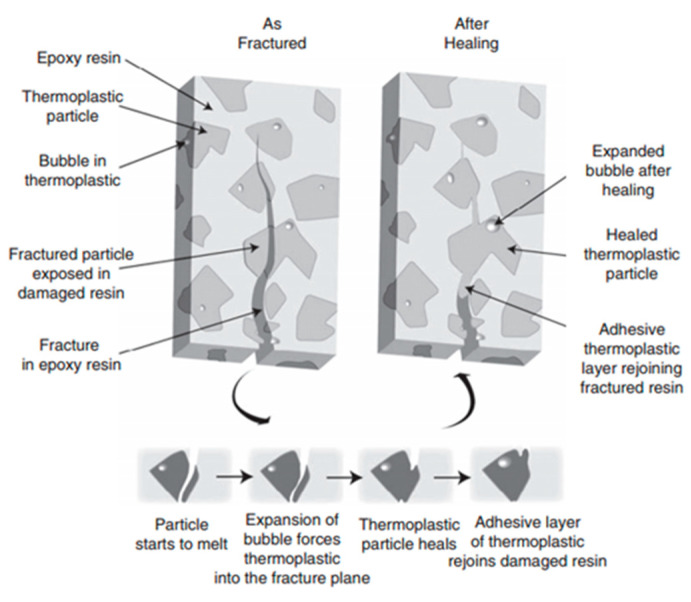
Healing agent delivery mechanism used by the mendable epoxy resins containing EMAA particles (reproduced from [57] with permission from Elsevier).

Moving towards thermoset/thermoplastic immiscible blends, considerable attention has been drawn to the use of EMAA as a cheap and efficient solid-phase healing agent. In the pioneering work of Meure et al. [57], EMAA was processed via continuous fiber extrusion and incorporated either as discrete particles (CFRP_p_) or as a fiber mesh (CFRP_f_), to design a carbon fiber-reinforced epoxy composite. Even though SEM imaging revealed different surface structures between the CFRP_p_ and CFRP_f_, both configurations were capable of completely restoring the peak load after healing at 150 °C for 30 min, yielding healing efficiencies over 100% in terms of mode I interlaminar fracture toughness, fracture energy, and peak load, providing in particular an astounding 221 ± 17% of energy to failure recovery for CFRP_p_. Healing was proven to occur via a pressure delivery mechanism of the healing agent [58]: EMAA flowing into a crack plane during thermal activation was reported to occur due to the expansion of small bubbles that facilitate healing as the particles rebind together the adjacent epoxy fracture surfaces (Figure 4).Varley and Parn studied the efficacy of non-woven EMAA fabric embedded within a carbon fiber epoxy composite via mode I and mode II failure testing [59]: fracture toughness and load were restored close to or over 100%, while flexural modulus was restored by about 80%.

Varley and coworkers also proposed an alternative two-step curing to design a high-temperature carbon fiber reinforced epoxy composite incorporating EMAA [60]: a preliminary step of curing below melting point of EMAA, i.e., 85 °C, followed by a long-time, high-temperature curing process, was needed to ensure the solid and immiscible state of EMAA during the whole cure regime, otherwise it would have experienced degradation. During this additional cure step, non-covalent hydrogen bonding and ionic associations bind the epoxy resin and amine to the EMAA surface, facilitating interfacial condensation reactions at higher cure temperatures later in the cure by shielding or encapsulating the EMAA carboxylic acid groups from unwanted epoxide reaction earlier in the cure. This system provides one of the highest T_g_s (217 °C, from E′ onset from DMTA tests), and at healing temperature of 200 °C and 230 °C (30 min and 20 kPa of applied pressure), it exhibited high efficiencies (55% and 105%, respectively) thanks to the combination of lower EMAA viscosity, enhanced molecular mobility, and better adhesion to the epoxy network. Furthermore, a relatively great amount of GIC recovered (~38%) was still achieved during healing at 150 °C, despite the epoxy network being in its glassy state. The same procedures have been employed to investigate a CFRPs with a DGEBA/DETDA matrix [61]: EMAA particles distributed evenly on the surface of the crack plane were found to enhance mode I interlaminar fracture toughness by up to 200%, and healing efficiencies of 82% and 114% were achieved by heating at 150 °C and 200 °C, respectively.

Another interesting approach to trigger healing behavior was explored by Wang et al. [62]: exploiting ultrasonic welding, repair efficiencies of mode I interlaminar fracture toughness up to 130% for laminates containing high concentrations of EMAA were achieved.

Some alternative immiscible thermoplastics have been explored as potential healing agents in cured epoxy resins and carbon fiber-epoxy composites [63,64]. Like EMAA, PEGMA revealed excellent healing behavior even after multiple healing cycles due to the pressure delivery mechanism described above. On the other hand, healing with EVA was due to favorable rheological flow and a highly elastomeric response to damage [63]. However, EVA is not better than EMAA in terms of healing efficiency when GIC tests on CFRP composites were carried out [64]. PEGMA, instead, showed only partial recovery (57%) to the mode I interlaminar fracture toughness of the composite following healing. Studies incorporating two types of thermoplastics, EMA and EMAA, have been carried out by Wang et al. [65]: carbon fiber composites with thermoplastic patches being placed between plies improved fracture toughness but reduced interlaminar strength, yielding healing efficiencies of 88% for EMAA and 46% for EMA.

Table 2 summarizes all the work on epoxies and epoxy composites detailed above, based on self-healing mechanisms via physical interactions.

### 3.2. Supramolecular Polymers

Supramolecular self-healing polymers rely on the formation of networks through the adoption of non-covalent bonds able to connect and reconnect via reversible “sticker-like” behavior [9]. The reversibility is provided in most cases by (i) hydrogen bonds; (ii) π-π stacking; (iii) ionic bonds; and (iv) metal-ligand interactions [38].

Upon mechanical stresses, the weaker supramolecular bonds break first, with the newly generated interfaces containing sticky groups that can recombine to heal the material. Despite their high dynamic properties that make them appropriate for designing self-healing systems, many aspects, mainly related to the timescale of the healing dynamics, are still unclear. In the context of epoxy monomers, very few supramolecular studies in the literature have resulted in relatively poor mechanical performance and/or low T_g_s [66,67,68,69,70], thus they are not very suitable for high-end structural FRP composites for aerospace structural applications.

### 3.3. Chemical Interactions

The general concept of dynamic covalent chemistry in self-healing polymers, related to dynamic covalent bonds which are formed in a reversible manner under conditions of equilibrium, was highlighted in 2002 in a groundbreaking review by Rowan et al. [71] As these bonds are subjected to an external stimulus, they become reversible and reach a state of equilibrium.

Dynamic covalent bonds have been successfully used in polymer synthesis, especially for controlled radical polymerization reactions [72], but they have also been employed to develop the so-called dynamers (or dynamic polymers), which include non-covalent interactions and/or dynamic covalent bonds, allowing a continuous modification in constitution by reorganization and exchange of building blocks, even after polymerization [28], providing self-healing features. In the context of thermoset polymers, the other great breakthrough in studying the makings of reversible covalent bonds is represented by covalent adaptable networks (CANs), which combine the desirable properties of thermosets with the dynamics of triggerable bonds, so that a microscopic mechanism could provide macroscopic flow and stress relaxation [73,74,75]. In other words, whichever trigger is imparted to a CAN, it results in a decrease in viscosity that makes the polymer amenable to flow yet preserving the total bond and crosslinking density. CANs are broadly divided into (a) dissociative CANs, where a new bond is formed after an existing bond is broken; (b) associative CANs, where cleavage of the existing bond occurs simultaneously with respect to the formation of a new one.

#### 3.3.1. DA/rDA Chemistry

**Figure 5:**
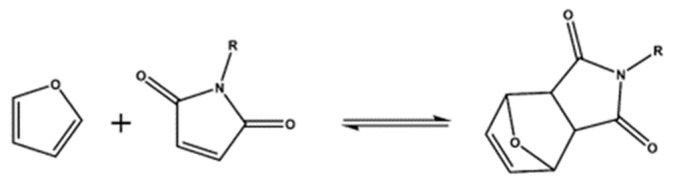
DA/rDA reaction between a furan group and a maleimide group.

Probably the most well-known chemical reaction used for intrinsic self-healing materials and a typical example of dissociative CAN formation is given by the Diels-Adler reaction (DA), consisting of a [4 + 2] cycloaddition between a diene and a dienophile: the formed DA adduct is thermally unstable and could experience a retro-DA (rDA) reaction at higher temperatures. Figure 5 shows the DA reaction between furan and maleimide groups.

The first patent on thermally reversible polymeric networks containing DA reaction was reported by Craven in 1969 [76], but the use of furan-maleimide systems for efficient self-healing was presented thirty years later by Wudl and coworkers [77,78].

However, since the incorporation of the DA group into epoxy monomers would be expensive to implement on a large scale, it is convenient to be able to use common, commercially available epoxy formulations and introduce DA adducts in other ways. One example is shown in the works of Palmese et al. [79,80]: a traditional self-healing epoxy-amine thermoset was mended with the addition of a reversibly cross-linked resin with furan and maleimide groups as a healing gel that is inserted after the occurrence of damage [79]. The relatively low healing efficiency of this system (37%) has been improved up to 70% by incorporating bismaleimide (BMI) dissolved in DMF, while the furan groups were introduced in the synthesis of the epoxy backbone [80]. This procedure is limited from a practical perspective due to the use of solvents, encapsulated reagents, and long healing times.

In another strategy adopted by Bai, Saito and Simon, the thermally reversible DA unit was incorporated into the diamine cross-linker [81]: although no healing efficiencies have been reported in this study, scratches on the DGEBA-based epoxy polymer disappeared after treatment at 140 °C for 30 min, with no reduction of healing potential experienced after numerous healing cycles.

From the publications on successful self-healing of polymers embedding DA adducts, it is evident that their major disadvantages are poor mechanical and thermal properties, especially when multiple-healing cycles are considered [39]. In an attempt to overcome these limitations, but also to improve the overall healing efficiency, Turkenburg and Fischer reported a two-step process in the synthesis of DA-based epoxies in order to avoid unwanted side-reactions that would cause the formation of irreversible crosslinks and compromise healing [82]. The obtained material was able to sustain multiple self-healing thermal cycles at 150 °C, 5 min for each cycle. This formulation has been further investigated by Coope et al. with a varying BMI content combined with commercial aerospace-grade epoxies, both in form of bulk material and thin film [83]: self-healing efficiencies reached values greater than 100% in some cases. The great feature of this system is its ability to be extruded and processed into a stable thin film that can be machined to a specific geometry to be interleaved or infused to form a pre-preg material. In addition, the fast healing procedure, which can be carried out by an external heating apparatus, could really make in situ repair possible.

Moving towards DA/rDA-based polymers as matrices in composites, a more comprehensive and detailed approach is required, especially at the matrix/reinforcement boundary that constitutes the weakest part of the material. The work of Peterson et al. [84] has focused on the introduction of DA adducts into the interphase of glass fiber-reinforced composites with epoxy matrices embedding various furan concentrations. Microdroplet single fiber pull-out tests provided an average 41% healing efficiency within the interphase, with no apparent differences with varying furan content. Martone and coworkers implemented an acidic chemical treatment at the interphase between glass fiber and matrix to promote rDA reactions with a two-step thermal healing treatment [85], while Zhang, Duchet and Gérard introduced DA bonds formed between maleimide groups grafted on a carbon fiber surface and furan groups introduced within the epoxy–amine matrix in the interfacial region [86]. Due to the location of these reversible bonds, the carbon/epoxy interphases self-heal with an efficiency up to 82% in the first healing cycle.

As previously stated, self-healing via DA reactions has been proven to be effective, yet the healing kinetics are usually slow, especially for high-Mw polymers, which feature high viscosity and low chain mobility. With this regard, Sun, Zhang and coworkers designed a rapid self-healing and recyclable high-performance crosslinked epoxy resin/graphene nanocomposite [87]: the presence of the multiple-responsive graphene allows for rapid self-healing not only through heat, but also via IR and microwaves. More recently, Chen, Shuai et al. designed a novel, flexible (elongation > 100%) self-healing epoxy material, fabricated with graphite nanosheets (GNSs) and an epoxy polymer, that can be mended by IR light [88]: healing efficiencies above 90% were achieved from lap shear strength tests after samples had been repaired via IR light absorbed in the epoxy composite samples by GNSs, which act as a nanoscale heater thanks to its excellent thermal conductivity.

Most recent efforts in DA-based self-healing formulations include integration of MWCNTs: Handique and Dolui developed a novel strategy for fabricating recyclable and repairable MWCNT-epoxy composites, that yielded almost 80% healing efficiency without significantly losing integrity and load bearing ability over repeated healing cycles [89].

Despite the vast literature on the topic of DA/rDA chemistry for self-healing purposes, the first report on DA-based healing of epoxy polymer considering not only interface healing between filler and matrix, as the abovementioned studies, but also across the bulk epoxy matrix system has not been published until very recently in 2019 by Khan and coworkers [90]: thermo-reversible self-healing nanocomposites were prepared by B-GNPs in a polymer matrix hybridized with thermo-irreversible DGEBA epoxy resin, responsible for the structural integrity of the system, and thermo-reversible DA-based resin system. The optimized weight ratio for the best trade-off between mechanical and healing behavior showed healing over five cycles without significant degradation of their mechanical properties, although always below 100% healing efficiency. Therefore, there is plenty of room for further improvement, not solely related to efficiency, but also focusing on easier and faster material processing techniques.

#### 3.3.2. Alkoxyamine and Imine Exchange

Among dynamic covalent bonds that have been reported to add self-healing functionality in epoxy systems, alkoxyamine exchange, imine exchange, disulfide exchange (Section 3.3.3 and Section 3.3.4), and transesterification (Section 3.3.5) are reported here. Yuan, et al. took advantage of simultaneous covalent bond homolysis and radical recombination of C-ON bonds to introduce self-healing attributes [91]: tensile and bending tests on healed (90 °C, 1 h, in argon), previously impacted EP/DGEBA blend yielded healing efficiency up to 62%. The advantage given by these catalyst-free, single-step alkoxyamine moieties over two-step healing strategies (e.g., DA bonds) is that material deformation due to collapse of crosslinked network while self-repairing is avoided, even when healed above T_g_. However, the synthesis route is rather laborious and difficult to implement on a larger scale. Furthermore, the reversible reaction of C–ON bonds cannot proceed in air due to the oxygen-induced deactivation of radicals: Zhang et al. managed to bypass this criticality by adopting a new synthesis procedure that assured oxygen insensitivity for healing at room temperature, achieving 60% of tensile strength recovery [92]. Although this may seem a favorable self-healing system and a step forward with respect to [91], the sub-ambient T_g_ evidently limits its range of applicability in the aerospace industry.

Reports on polymeric networks embedding imine linkages are limited. A very recent study by Zhao and Abu-Omar may be interesting for the purposes of this review [93]: imine bonds exchange in a vanillin-based, bisphenol-containing recyclable thermoset exhibits fast relaxation times at relatively low temperatures. This permits degradability, recyclability, malleability and weldability without requiring metal catalysts or additional monomers.

#### 3.3.3. Dynamic Sulfur Chemistry

Another specific subset of dynamic covalent chemistry is playing a crucial role in the development of self-healing materials: the use of sulfur–sulfur reversible covalent bonds for the generation of dynamic systems is referred to as dynamic sulfur chemistry and it represents an important class in dynamic covalent chemistry [94]. Sulfur moieties are of interest in self-healing systems because disulfide bonds are weaker than carbon–carbon bonds, thus their mechanical scission is much easier [95]. Examples of dynamic sulfur systems are reported in the literature, such as transthioesterification [96], thioacetal exchange [97], conjugate thiol addition [98], and even the more widely studied thiol/disulfide exchange [99].

Although in principle any molecule or polymer where at least one disulfide bond is present can be envisaged as a potential candidate for self-healing formulations, higher bond reshuffling rates (i.e., faster healing) can be achieved with tetrasulfides, thanks to their lower stability with respect to tri- and di-sulfides. The work of Lafont et al. on thermoset networks containing disulfide bonds demonstrated that healing kinetics is influenced by the number of thiol functions embedded in the crosslinker [100]: the produced materials exhibit full cohesive recovery in 20–300 min at 65 °C, depending on the physical properties of the different formulations. Adhesive strength tested on Al alloys is fully recovered, even after multiple failure events.

Despite the aforementioned advantage derived from dealing with tetrasulfides, a major drawback for their presence is represented by a decreased mechanical and thermal stability of the material. In order to overcome this limitation, hybrid organic–inorganic crosslinked networks containing also non-reversible crosslinks, which provide mechanical robustness, have been studied. The effect of crosslinking density and content of tetrasulfide groups on the initial mechanical properties on ~600 μm-thick films have been considered, as well as their temperature-dependent gap closure kinetics [101]. The higher crosslinking density due to increasing the inorganic and the organic crosslinks led to an increase in toughness and stress at break, with the inorganic portion having the largest effect. Gap closure efficiency, which has been proven optimal at 70 °C, is lowered by increasing the rigidity of the network, at constant values of reversible bonds. Nevertheless, the highest gap closure efficiency does not correspond to the lowest mechanical properties when considering samples containing different contents of reversible groups, meaning that the controlling factor on the healing performance of the hybrid sol–gel films is indeed the content of tetrasulfide groups. Furthermore, interfacial strength recovery on SENT specimens has been studied: the healing treatment (70 °C for 2 h in an air circulation oven) resulted in a recovery of about 70% of the failure load. Further analyses have been conducted to identify the effect of curing time on the polymer bulk properties, its interfacial healing performance, flow and fracture by comparing polymers cured for 2 and 48 h [102]. Longer curing times provide a more crosslinked, and thus stable, polymeric network with improved yield stress (about three times higher than that of short curing times) and a reduced plastic region, indicating a less ductile behavior and a reduced flow tendency, in-line with the higher E_a_ found for the highly crosslinked polymer via rheological measurements. Interestingly, not only the maximum interfacial healing efficiency (~60%) was found to be independent of the curing degree, but also, for short times, the healing efficiency of the more rigid 48 h-cured polymer was higher than that of the more ductile, 2 h-cured one. This is due to the higher interfacial wetting facilitated by the formation of a smoother surface during the fracture process of the longer-cured, and thus more brittle, polymer.

Research on a glass fiber-reinforced polymer composite based on a disulfide-containing organic–inorganic thermoset matrix has been carried out by Post et al. [103]: after an optimization analysis in terms of thermomechanical properties and curing regime, the composite has been proven to almost totally heal small scale sized damages (<cm^2^) from low-velocity impacts by applying minimal pressure at moderate temperatures (70–85 °C).

#### 3.3.4. Aromatic Disulfide Exchange

The exchange of disulfides has been reported to occur via catalysts, mild temperatures or UV radiation at room temperature [94]; but to design a polymer network capable of healing at room temperature without the need for any stimulus, aromatic disulfides, whose exchange mechanism had been reported to occur at room temperature [34], were proposed instead of aliphatic counterparts.

**Figure 6:**
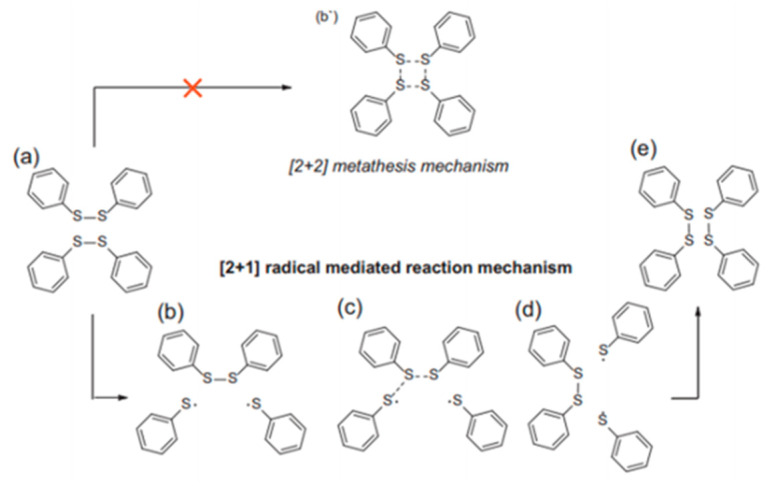
Schematic representation of a [2 + 2] metathesis (above) and a [2 + 1] radical mediated (below) reaction mechanisms [105].

For aromatic crosslinks, both [2 + 2] metathesis and [2 + 1] radical-mediated mechanisms have been studied in the literature to describe how disulfide bonds break and re-form (Figure 6). In the first case, the disulfide bonds would break and form simultaneously, while in the radical-mediated mechanism the breaking of one disulfide bond would lead to the formation of sulfur-centered radicals that would eventually attack other disulfide bonds. Some works have been published with both mechanisms used indistinctly to describe the exchange reaction, while other researchers have described the disulfide exchange without specifying the underlying mechanism. Given that, further analysis of the mechanisms involved was carried out by Matxain et al. [104]: no computational evidence was found for the existence of a metathesis transition state, thus a radical-mediated [2 + 1] mechanism was proposed for the dynamic behavior in aromatic disulfide systems.

Among the aromatic disulfide-containing agents, the most popular one is probably 4-aminophenyl disulfide (4AFD, sometimes referred as DTDA for 4,4′-dithiodianiline): Tesoro and coworkers first studied the feasibility of reversible crosslinking in 4AFD-cured epoxy resins in the late ‘80s [105,106,107], Johnson et al. studied the ability to control the degradation of disulfide-containing epoxy materials by means of a specific thiol trigger, i.e., 2-ME [108]. Degradation profiles were controlled by tuning temperature, stoichiometry of monomers and quantity of disulfide groups. This latter work showed the recovery of only the reinforcement rooted into a disulfide-based epoxy composite. Otsuka and coworkers have reported a new degradable epoxy resin prepared by the reaction of epoxy monomers containing S–S bonds with different amine curing agents [109], indicating facile degradation within tens of minutes.

**Figure 7:**
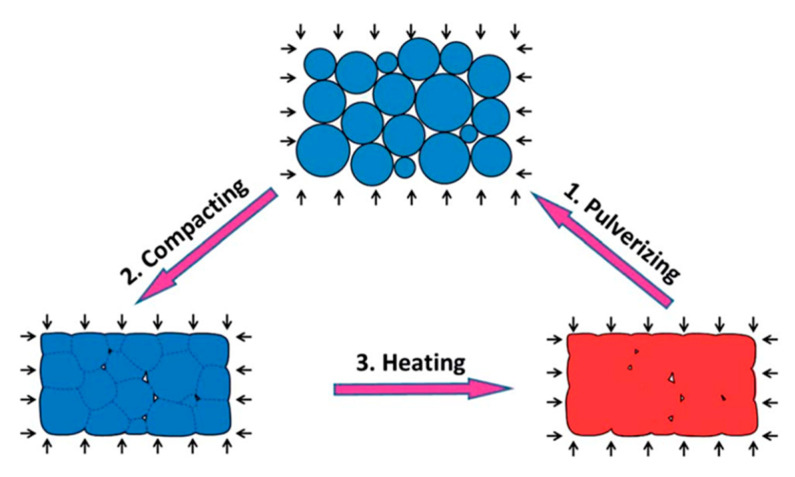
The schematic graphs for a typical reprocessing and recycling routine. (**1**) Bulk polymer is firstly pulverized into powders. (**2**) The powders were then compacted by applying pressure. (**3**) The compacted powder is heated under pressure. Due to the internal bond exchange reaction (BER), the polymer particles are welded, and interfaces are disappeared. This process is repeated several times (reproduced from [110] with permission from Royal Society of Chemistry).

Chemical functional groups that degrade in response to chemical or thermal triggers are of paramount importance not only for specific applications in extreme environmental conditions, but also in the context of material reprocessing and recycling [108]. Degradation leading to reprocessing and recycling of epoxy networks and epoxy matrix composites can be carried out with the pulverized material (Figure 7), so that small-sized polymer particles could be easily handled, transported and stored, as pointed out by a communication from Yu et al. [110], which also detailed the effect of reprocessing conditions (e.g., time and pressure) on mechanical performances.

#### 3.3.5. Transesterification

A novel high-performance thermosetting epoxy polymer/chain-extended bismaleimide (EP/CBMI) system with dynamic transesterification bonds has been reported by Ding et al. [111], yielding relatively high-T_g_ polymers (up to 125 °C) with excellent shape memory cycle stability. The system is able to quickly recover its original shape within 21 s at 150 °C, while, as far as healing efficiency is concerned, *η* values for first and second cycle of fractured DGEBA/CBMI could reach from 78 to 87% and from 75 to 84%, respectively, varying with increasing CBMI concentrations.

The combined effect of shape memory and transesterification reaction has been further analyzed by Li with DGEBA and tricarballylic acid [112]: the healing process, achieved by compression programming two saw-cut epoxy block specimens stacked in a confined space at 150 °C for 18 h, yielded a value of about 60% for the worst-case testing scenario (i.e., crack lines not matched or aligned during healing).

Recyclability was the focus of Lu et al. in the design of a DGEBA-phthalic anhydride system [113], where a healing efficiency as high as 88% was measured via uniaxial tension tests. This study pointed out that recycling is affected by several parameters, not only temperature and pressure but also particle size (recycling achieved by ball milling, obtaining powdered material), particle size distribution and milling time. These works underline that healing by transesterification is highly dependent on the presence of reactant groups (typically DGEBA), catalysts, and the reaction conditions.

#### 3.3.6. Vitrimers

A huge stride forward in the design of self-healing epoxy networks has been taken with vitrimers, a remarkable example of associative CANs that have been widely investigated for the last few years. Vitrimers consist of a covalent organic network that can rearrange its topology via reversible exchange reactions that preserve the total number of network bonds (i.e., crosslinking density) and the average functionality of the nodes [114].

The term itself refers to their “vitreous-like” thermal behavior observed for the first time by Leibler et al. for epoxy systems containing β-hydroxyester reversible moieties [115]. Exchange reactions in vitrimers normally are triggered by heat: at high temperatures, viscosity decreases with an Arrhenius-like law and fast exchange reactions enable stress relaxation and malleability, and upon cooling, the exchanges become so slow that the topology of the network is essentially fixed and the system behaves like an elastic thermoset (i.e., an elastomer). This allows the vitrimer thermosets to be reprocessed using conventional thermoplastic processing techniques, e.g., injection and compression molding, extrusion, 3D printing, etc. As vitrimers maintain permanent networks with a permanent connectivity at all temperatures (until degradation phenomena occur), these materials swell but do not dissolve in chemically inert solvents, even when heated [30].

The viscoelastic behavior of vitrimers is described not only by T_g_ but also by the so-called topology freezing transition temperature, denoted as T_v_. When the timescale of bond exchange reactions becomes shorter than the timescale of material deformation, the network can flow and rearrange, thus a transition from viscoelastic solid to viscoelastic liquid occurs. This transition temperature is conventionally chosen at the point where a viscosity of 1012 Pa·s is reached [116]. For the design of vitrimer materials, it is important to consider the two transitions and their corresponding temperatures, T_g_ and T_v_, which can be controlled through parameters such as crosslinking density, intrinsic rigidity of monomers, exchange reaction kinetics (e.g., catalyst loading) and density of exchangeable bonds and groups.

Since the publication of the abovementioned seminal paper by Leibler, many transesterification vitrimer thermosets have proven to be suitable for mass production [117,118,119,120,121], thanks to high availability of monomers and ease of synthesis. However, fast processing is only achieved with high amounts of catalyst, whose insolubility is also detrimental to the design of high T_g_ materials (>75 °C), which is indeed the temperature range useful for structural aerospace applications.

To provide self-healing functionalities in vitrimer-like networks from already commercially available precursors, without the need for catalysts, aromatic disulfide exchange constitutes a valid alternative. Advanced work in the design of epoxy vitrimer composites has been done by Odriozola and coworkers [122,123]: DGEBA epoxy resin cured with 4AFD yielded a relatively high T_g_ (130 °C) polymer with very fast relaxation times (from 3 h at 130 °C to 20 s at 200 °C). Reprocessing and recycling of the epoxy network was assessed by hot press experiments at 200 °C and 100 bar for 5 min: powdered material was reshaped in the form of a film that showed the same mechanical properties as the pristine sample. Repairing a small scratch on the material was succeeded by applying heat and pressure with a household iron at 200 °C. Furthermore, reprocessing ability of multilayered dynamic carbon-fiber reinforced composites was successfully tested by hot pressing, whereas quantitative repairing and strength recovery was achieved on glass-fiber reinforced composites, as assessed by interlaminar shear strength tests. Chemical recycling of fibers was successful via epoxy matrix dissolution with 2-ME in DMF, whereas mechanically recycled second-generation composites were obtained by grinding FRP scraps into powder and subsequent compression molding.

**Figure 8:**
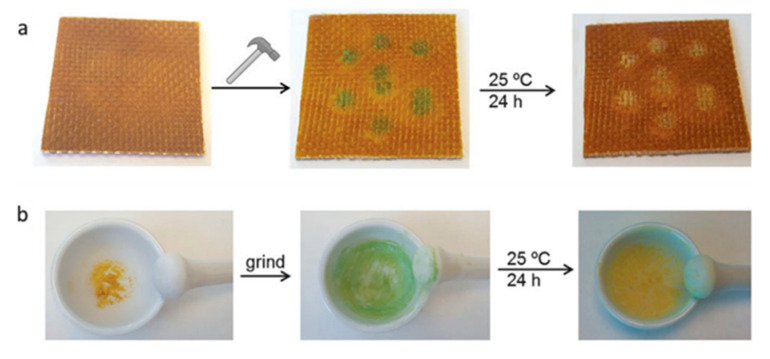
(**a**) Photographic sequence showing the glass-fiber composite being hit using a hammer, which resulted in the appearance of green spots, and, after 24 h at room-temperature, the green color disappeared. (**b**) Photographic sequence showing how cured epoxy vitrimer in the form of powder became green after grinding in a mortar. After 24 h at room-temperature the green color disappeared (reproduced from [123] with permission from Royal Society of Chemistry).

This system is endowed with a remarkable added functionality, consisting in the inherent ability of showing a transient green coloration after a damage event (Figure 8). This phenomenon, known as mechanochromism, occurs either when the composite is machined (grinded) or hit by a hammer and is supposed to be due to the formation of sulfenyl radicals, promoted by the mechanically assisted excision of the aromatic disulfide crosslinks [123]. Such radicals, being almost immobile at temperatures below T_g_, could survive several hours before pairing up to form new bridges, hence the transition disappearing in 24 h at room temperature and in less than 30 s when heated at 150 °C (i.e., above T_g_), while the coloration is maintained for several days when specimens are kept at −20 °C.

As the synthesis of the epoxy system in the works of Odriozola was carried out in excess of 4AFD, Paolillo and Grande recently investigated the abovementioned functionalities with the formulation at stoichiometry [124]: inherent mechanochromic behavior, reprocessing from powdered material and adhesion tests have been confirmed in the formulation at stoichiometry, which possesses a remarkably higher T_g_ (170 °C) and a stiffer network.

4AFD was also used in the synthesis of a CNT/polypyrrole/vitrimer composite (CPV) by Zhang and coworkers [125]. The relevance of this study resides not only in its findings, but also in its versatile synthesis strategy, which can be adopted by incorporating other monomers depending on specific required properties. Furthermore, this double-disulfide-embedding dynamic epoxy matrix network showed slightly higher T_g_, faster relaxation times and lower activation energy with respect to other vitrimers reported in the literature. The presence of minimum 0.5 wt% highly dispersed CNT/polypyrrole increased the conductivity of the composite via percolation, making CPV a great candidate for electromagnetic shielding and microwave absorption. Mechanical reprocessing by hot pressing of CPV in powder form with no significant losses in electrical performance has been successfully verified. In addition, complete degradation of the resin could be achieved in DMF solution of dithiothreitol (50 °C for 24 h), allowing for the recovery of CNT/polypyrrole filler.

#### 3.3.7. Bio-Vitrimers

Based on publicly accessible data, about 10–15% of total plastics production volume is covered by thermoset resins [126], but their global market size is forecast to grow by 40% in the upcoming decade [127]. Increasing usage of thermosets will in turn result in an increased amount of waste and higher demand for recycling options, which currently present many limitations especially in the aerospace sector. In addition to the environmental pollution generated by waste disposal, the depletion of petroleum resources has propelled the academic landscape towards biomasses as valid alternatives to fossil-based formulations.

In line with the theme of sustainable and circular economy, we mention below some recent examples of bio-based epoxy vitrimers with comparable thermomechanical properties with respect to similar epoxy networks. Zhang and coworkers [128] designed and synthesized TEP by using renewable lignin-based vanillin and guaiacol, which has been cured with an anhydride to obtain a bio-vitrimer with a range of remarkably high transition temperatures (tunable by varying TEP content, nevertheless the maximum T_g_ exhibited was 187 °C). Experiments on the repairing of scratches on film surfaces have shown crack width reductions of over 70% in just 5 min at 220 °C. However, performing the repair for longer times could result to be detrimental because of the high operating temperature (thermal degradation will occur at t > 10 min). In the works of Liu et al. [129], a polymer network with above-room temperature T_g_ consisting of ESO cured with FPA was able, through transesterification reactions, to rearrange its structure by heating, providing self-healing and shape memory features, as well as controlled degradation for reprocessing into new vitrimers.

As described in the previous section, the need for catalysts in transesterification-based rearrangeable networks constitutes a limiting factor in the design of vitrimers: bio-based formulations are no exception. Rapid metathesis of disulfide bonds at T > T_g_ represents a valid alternative, which has been explored by Wang and coworkers with a bio-based vitrimer constructed from isosorbide-derived epoxy and aromatic diamines [130]. The system permits total healing of surface cracks (100 °C for 1 h), multiple recycling and reprocessing of powdered material (hot press at 100 °C for 1 h), as well as controlled degradation in alkaline aqueous solutions, and also exhibits a shape-memory effect.

Finally, Table 3 compiles all the references on intrinsic self-healing epoxies and epoxy composites via chemical interactions.

## 4. Discussion and Application Perspective

**Figure 9:**
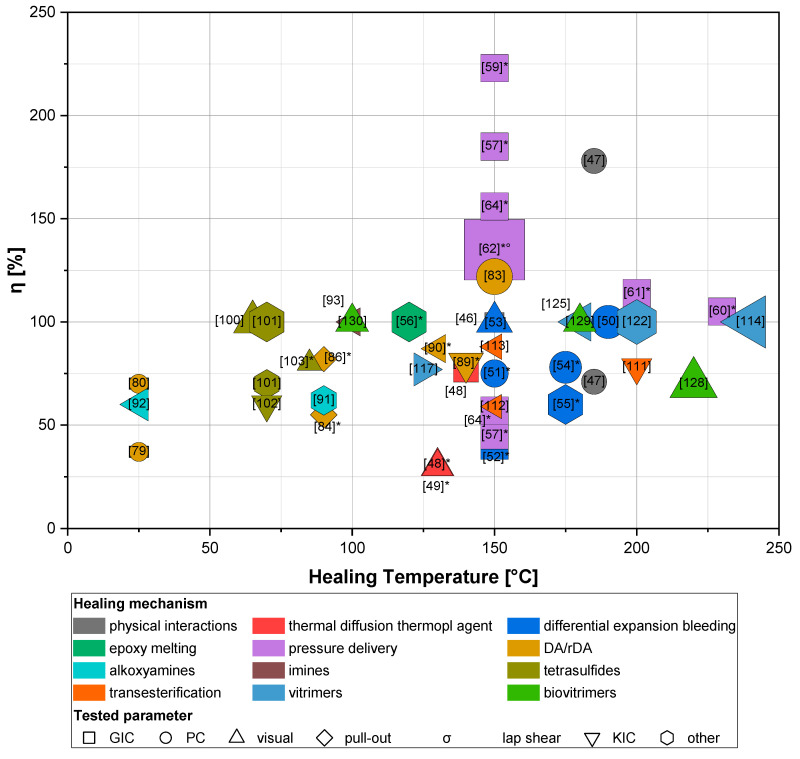
Graphical representation of the intrinsic self-healing formulations described in this review in terms of healing temperature and healing efficiency, with color coding for healing mechanism, shape coding for tested parameter adopted for assessing healing efficiency, and area inversely correlated with healing time (i.e., bigger area = shorter times). If ‘*’ next to [ref.] is present, it indicates that the material is a composite. Otherwise, it is an epoxy as a standalone.

In this section, we aim to give an overview of the field and summarize the exhaustive literature on this topic. Given that there is no “gold standard” in testing protocols or quantifying self-healing, we attempt here to compare the results reported using various polymer chemistries and using vastly different test methods. Figure 9 shows an overview of the literature examples described in Section 3 and their relation between healing efficiency and healing conditions. Since the majority of said examples employ a thermal trigger (except for those healed with IR and/or microwaves [87,88,119], which have been not included in the plot), the healing temperature is presented as the *x*-axis. Duration of healing is represented by the area of the symbols in an inverse fashion, meaning that bigger symbols correspond to shorter healing times, i.e., the slowest healing, thus the smallest marker area and the fastest, thus the biggest one. The analyzed healing time ranges from seconds (30 s [62]) to hours (25 h [86]). In addition, symbols are color-coded depending on the healing mechanism and their shape refers to the parameter that has been tested to assess *η*.

At first glance, the combination of healing efficiencies above 100% and elevated healing temperatures (which are symptoms of high T_g_s) may led one to assume that the top-right portion of the plot is indeed the target region for intrinsic self-healing epoxy FRPs in order to be adopted in aerospace applications. Yet, other factors have to be considered: mechanical properties, for example, play an important role in FRPs for their suitability in structural applications.

Take the DGEBA-PACM system studied [47]: the formulation in excess of epoxides surely aided the increase in fracture load of healed specimens, giving a 178% healing efficiency by facilitating a mechanism of covalent bond formation of unreacted groups at a crack interface. However, fracture load of pristine samples had almost halved in relation to the formulation at stoichiometry, which yields a more modest 71% of peak recovery. Furthermore, the stability of non-stoichiometric systems needs to be carefully addressed for aerospace applications, where a prolonged lifetime of the components is required.

Relatively poor healing performances have resulted from epoxy/poly(bisphenol-A-co-epichlorohydrin) systems (symbols in red, [48,49]). Furthermore, the upper service temperature of the blended FRPs limited to the melting temperature of the added thermoplastics (i.e., 160 °C) makes their use critical in space environments and high temperature aeronautic applications, yet still employable for low and moderate temperature aeronautic components. However, epoxy/poly(bisphenol-A-co-epichlorohydrin) blends were somewhat important from a historical perspective. In fact, when the work of Hayes et al. [48] was published, few tailored matrix systems designed to self-heal via chemical interactions were employed and studied, mainly because of the need for completely new matrices and manufacturing technologies. Hitherto, despite the promising features of self-healing via chemical routes, it was yet to be proven whether materials would have had sufficient mechanical properties to make them attractive as alternative matrices in PMCs for the aerospace field. For these reasons, Hayes’s solid-state healable system based on conventional thermosetting resin technology had been successful in the infancy stages of this field of study. To the present day, self-healing via chemical interactions has been extensively studied and implemented in commercially available epoxies and common manufacturing practices, paving the route to real aerospace applications.

Despite a lower Tm with respect to poly(bisphenol-A-co-epichlorohydrin), PCL gives epoxy blends whose T_g_s reside more to the higher-end of the spectrum. The great advantage of such systems (represented by the dark blue markers in Figure 9 [50,51,52,53,54,55]) is the homogeneity of the blend after curing, which makes them compatible with processing techniques for fiber-reinforced composite manufacturing, provided that its viscosity does not increase with blending [51]. In addition, strong bonding between two pieces of material has been proven successful: this noteworthy feature should not be underestimated because it can potentially lead to the production of complex geometries by manufacturing simpler shapes that could be eventually joined together, decreasing manufacturing complexity and therefore costs of aerospace components.

As far as immiscible blends are concerned [56,57,58,59,60,61,62,63,64], some practical aspects have to be taken into account: surely in principle cracks could be partially filled numerous times for the same fracture surface, but in reality, the damage can only be filled up proportionally to the added volume fraction of healing agent [35], and healing can never be as complete as depicted in the ideal cases presented in the literature. Furthermore, it was also reported that voids formed after the embedded healing agents flowed into the damaged location negatively affected the integrity of the material [56], in particular fatigue performances may dramatically decrease. Nevertheless, much effort has been put into developing these systems, in particular those incorporating EMAA pellets with a two-step curing procedure [60,61], which yielded thermally sound carbon-reinforced composites.

It is worth mentioning the work of Wang et al. [62] for their alternative healing trigger by means of ultrasonic welding: this method provides the fastest healing time (30 s). This and other triggering practices [87,88,119] are testimony to the potential portability of perspective healing devices designed ad hoc for the rapid in-field repair of aerospace composite structures. For this to happen, though, an embedded sensing system capable of receiving the healing triggers is necessary [26].

Moving towards chemical routes to self-healing, DA/rDA chemistry has been widely investigated, yet poor thermomechanical properties and slow healing speeds (especially for high-Mw polymers) limit their use over the interested range of temperatures. On the composite side, different surface modification techniques have been adopted to generate reactive groups onto the reinforcement, to increase self-healing ability at the matrix/reinforcement interface [39]. Alas, very few studies have dealt with healing of the bulk polymer matrix phase (we reported one example in this review, i.e., [90]).

Dynamic exchange mechanisms such as alkoxyamines, imines and tetrasulfides show lack of thermomechanical stability at high temperatures. The examples described in Section 3.3.2 present many issues related to the unfeasibility of healing processes in the air due to the occurrence of undesired side reactions, thus complicating the overall service and maintenance regime of the material, and insufficiently high transition temperatures (which is even sub-ambient in some cases, e.g., [92]). All these aspects are critical for aerospace applications and further studies are required in the future to overcome these issues prior to real application in aerospace composite parts.

Exploiting transesterification reactions appeared fruitful in obtaining self-healing formulations possessing great thermomechanical properties and healing functionalities, but, as already mentioned, a high amount of catalyst, which may result in a more arduous synthesis route, is necessary for fast processing. Furthermore, catalyst insolubility limits the design of high-T_g_ materials. This issue was bypassed with vitrimers, where the crosslinked epoxy is able to interchange fragments of the network structure: below the T_v_, transesterification is so slow that the vitrimer behaves like a conventional permanently cross-linked polymer network, and above T_v_, transesterification becomes accelerated and the network starts to flow like a viscoelastic liquid, yet keeping its integrity and number of covalent bonds [119]. Thus, it is important for the design of aerospace parts made of vitrimer materials to consider the two transitions and their corresponding temperatures, T_g_ and T_v_, which can be controlled through parameters such as the crosslink density, intrinsic rigidity of monomers, the exchange reaction kinetics (e.g., catalyst loading), and the density of exchangeable bonds and groups. For most applications, vitrimers should behave as classical thermosetting polymer networks in a useful temperature window, i.e., without significant creep [30], since it has been demonstrated that vitrimers can be designed with strongly suppressed creep and excellent reprocessability by incorporating a substantial yet subcritical fraction of permanent cross-links [131]. Nevertheless, in order to circumvent the issues related to the use of catalysts, vitrimer-like structures employing aromatic disulfide exchange (such as [122,123,124,125]) have been demonstrated to be a great alternative, with their higher transition temperatures and comparable if not improved thermomechanical stability. In addition, added functionalities, such as mechanochromism, could be beneficial in the monitoring and maintenance aspects of composites’ life cycle, all leading to diminishing costs from either faster inspections or less frequent maintenance of aircrafts.

Another way at striving for decreasing costs in the long run is the installment of a circular economy also for aerospace composites: even though the recycling of plastic materials is achieved only for a very small percentage of all plastics produced (in 2016 was 10% in average [132]), the recycling and reprocessing of thermosets may now become achievable with self-healing polymers, particularly those embedding exchangeable chemical bonds. Currently, most of the thermosets waste is being managed by landfilling, which proves to be very inefficient in extracting the maximum possible value from dismissed components. In addition, the currently used waste treatment techniques require high amounts of energy and rarely take recycling of the polymer matrix itself into account, focusing on regaining the more valuable reinforcing fibers. Therefore, the development of low-energy methods for re-obtaining the polymer phase in the form of monomers, oligomers, or thermoplastic polymers should be a focal point in upcoming years [133]. Theoretically, the most suitable formulations for also recovering the matrix material are thermosets with dissociative covalent linkages, where bonds can be broken while being stimuli-triggered in such a way that the drop in viscosity is sufficient for easy matrix removal [134]. In this respect, DA reversible linkages seem to be the most promising mechanism, but processing of DA functional groups into pre-pregs seems a challenging process. Despite many successful demonstrations of reprocessability and recycling reported in the literature, vitrimers, whose linkages are associative in nature, present difficulties in matrix-fiber separation, regardless of the exchange reaction involved. Further investigation is necessary, not only for merely monetary purposes, which drive the economy, but also for reducing carbon footprint and the use of petroleum-based resources in aerospace composites.

## 5. Conclusions

Intrinsic self-healing epoxy materials have gained much interest in the last two decades, finding potential applications in the aerospace field. The combination of several advantages from both inherent epoxy properties (great thermal stability and excellent mechanical properties, very good adhesion to substrates and fibers, and resistance to corrosion and moisture absorption) and added functionalities provided by physical and/or chemical self-mending mechanisms results in upgraded composite components, either structural or non-structural, with extended service life and new functionalities. The net benefit gained from the synergic effect of these two areas translates to (a) less inspection and maintenance times, i.e., an increase in the in-service/out-of-work ratio, as structures inherently possess what is necessary for healing damages; (b) a longer life cycle of any composite component; and (c) overall reduction of costs (maintenance, energy and even materials for some cases), as well as an increment of safety.

In this review, several examples of self-healing epoxies, either as standalone materials or, more importantly, those employed as matrix phase in composite materials, have been taken from the literature and described according to their repair mechanisms, the testing methods adopted to evaluate their healing efficiencies and their possible added functionalities that could emerge useful for aerospace applications. The review focused first on physical ways for healing, which were developed in the infancy stages of this field of study, but then moved towards more modern approaches involving chemical processes. The latter has appeared to be the more promising route. It has been highlighted how numerous variables have to be considered in designing self-healing epoxy formulations, e.g., chemical structure of the polymer backbone, its chain flexibility, the amount and the nature of the reactive sites that are responsible for healing. Many different chemistries have been described in this review, yet one concept appears crucial regardless of the chemistry employed: healing requires that local reversibility (i.e., bond forming-breaking reactions) is significantly faster than global processes (e.g., polymer flow and macroscopic deformation) to avoid total deformation of the material (shape loss) before healing [5].

Furthermore, this same concept is indeed valuable even when shape is lost: surely, it must not happen while in-service, yet total deformation opens the possibility of recycling not only the reinforcement, which is common practice in both academic and commercial environments, but also the matrix phase. Recovering thermoset polymer matrices has been reported to be difficult, especially in the aerospace field, where large composite parts are employed. However, a huge drop in matrix viscosity demonstrates in some self-healing epoxies provided by triggering the structure beyond the point of healing (but below the onset of degradation phenomena) could theoretically help to achieve such a feat.

Even when recycling is not feasible, reprocessing is a great way for economic savings and waste reduction: for example, in some cases it is possible to reshape composite parts and weld components together by exploiting the well-established adhesion properties of epoxies. Complex aerospace composite geometries, which typically require the use of long and costly manufacturing techniques (e.g., autoclave), could be assembled from easier, and thus cheaper to produce, flat panels that can be reshaped and chemically joined.

Nevertheless, some drawbacks arise when dealing with intrinsic self-healing formulations:

1. When dealing with some of the aforementioned healing mechanisms, fabrication of those polymers is rather complicated and costly. Tailor-made synthesis routes are unfavorable for the industry and for an upscaling of the fabrication process [135]. Thus, it is critical to implement these chemistries into well-established procedures adopted in the industry.
2. The healing processes described in this review are not fully autonomous, meaning that they requires an external energy source to trigger the healing mechanisms. In most cases, the trigger for the drop in viscosity is driven by an increase in temperature, which may not be seen as an issue in the academic domain, as testing is done on small samples of material. Nevertheless, it is foreseen to be quite cumbersome and expensive for large aerospace structures, however local heating strategies can be adopted to overcame this issue. A more thoughtful means of the temperature trigger could be envisaged with devices that supply heat, e.g., via IR, microwaves or ultrasounds, in a localized fashion. These methods could speed up even more the repair process, yielding even shorter maintenance times of aircrafts and spacecraft. Autonomous repair in intrinsic self-healing could be theoretically achievable by controlling the healing rate through combining energy delivery control and sensing [26].
3. Intrinsic self-healing is typically restricted to a small damage zone. Even if these materials can heal microcracks before any crack growth leading to catastrophic failure, thus de facto increasing the fatigue life of components. However, following high-energy impact, the material cannot heal when large damages are produced. When it does, it is generally because its size is relatively large (on the order of cm^2^). In the aerospace scenario, this may represent a major setback, since self-healing polymer matrices have always been associated with the idea of counteracting the deficiency of composites regarding impact resistance. This issue can be overcome by including into self-healing polymers a shape memory capability so that the shape memory will bring the fractured surfaces in contact and the intrinsic healing mechanisms will occur [136]. This so-called close-then-heal (CTH) strategy could also be an incredible help in bringing in contact fracture surfaces of load-carrying real-world structures [112].

Given the addressed issues, the range of applications is still vast, even if only uses for aviation and space are considered. Self-healing is one of the most promising approaches to design low-maintenance, lightweight composite fuselages. Self-healing polymer composites were also assessed for protecting space structures from space debris [137]. The ability of self-heal could be of paramount importance for critical pressurized modules and reservoir-type designs.

## Figures and Tables

**Table 1:** Examples of healing efficiency definitions based on the recovery of different material properties.

Material Property	*η* Definition	Notes
Fracture toughness	$\eta =\frac{K_{IC}^{H}}{K_{IC}^{V}}\times 100$	K_IC_ = fracture toughness (mode I)
Strength	$\eta =\frac{\sigma_{X}^{H}}{\sigma_{X}^{V}}\times 100$	σ = stress X = tensile, compressive, impact, flexural
Stiffness	$\eta =\frac{E_{H}}{E_{V}}\times 100$	E = Young’s modulus
Strain Energy	$\eta =\frac{U_{H}}{U_{V}}\times 100$	U = strain energy

H = healed, V = virgin.

**Table 2:** Summary of intrinsic self-healing formulations via physical interactions.

Healing Mechanism	Formulation	Healing Conditions	T_g_	*η* ^+^	Test (Specimen)	Notes	Ref.
			(°C)	(%)			
Micro-Brownian motion	Bisphenol A-based epoxy-NMA-BDMA	150 °C, 12 h	120	~100 *	G_IC_ (rectangular cast slab)	no clamping pressure required	[46]
Mech. interlocking	DGEBA-PACM	185 °C, 1 h, 8–13 MPa	162	71 ± 12	P_C_ (CT)	at stoichiometry	[47]
Mech. interlocking + polyetherification-homopolymerization			118	178 ± 56		excess of epoxy groups	
Diffusion of thermoplastic healing agent	DGEBA-based epoxy-NMA + poly(bisphenol-A-co-epichlorohydrin)	140 °C, 1 h	<74 *	77	G_IC_ (CT)	20 wt% thermopl. agent	[48]
				64	K_IC_ (CT)		
	*E-glass* + DGEBA-based epoxy-NMA + poly(bisphenol-A-co-epichlorohydrin)	130 °C, 1 h	74	>30	Visible damage area	10 wt% thermopl. agent	
		130 °C, 2 h		~30		optimized 7.5 wt thermopl. agent	[49]
Differential expansive bleeding	DGEBA-DDS + PCL	190 °C, 8 min, 18.7 kPa	203 ^	>100	P and U at failure (SENB)	15.5 wt% PCL, non-brittle behavior	[50]
	*CNFs* + DGEBA/DGEBF epoxy + PCL	175 °C, 10 min	72	78	peak bending P	10 wt% PCL, 0.2 wt% CNFs	[54]
			54–68	60	WOF		[55]
	DGEBA-DDS + PCL	150 °C, 30 min	197	70–80	P_C_ (TDCB)	25–26 vol% PCL	[51]
	*E-glass* + DGEBA-DDS + PCL	150 °C, 30 min	197^	82	slope of P-Δl (DCB)	25–26 vol% PCL, *η* increasing for subsequent healing cycles	[52]
				~40 *	G_IC_ (DCB)		
				100	CAI, C-scan (damaged area)	impact damage of 8 J	[53]
Epoxy particles	*Glass fiber* + coldsetting epoxy + thermosetting epoxy particles	120 °C, 10 min	n.a.	~100 *	stiffness (TPB)	nearly full recovery in rigidity	[56]
				>100 *	fatigue (SENT)	fatigue life extension	
Pressure delivery of healing agent	*CFs* + DGEBA-TETA + EMAA	150 °C, 30 min	n.a.	221 ± 17	failure energy (DCB)	15 vol% EMAA particles	[57]
				185 ± 26	G_IC_ (DCB)		
				137 ± 10	failure energy (DCB)	EMAA 2D fiber mesh, 4 interleaves	
				45 ± 9	G_IC_ (DCB)		
	*CFs* + DGEBA-TETA + EMAA	150 °C, 30 min	n.a.	223	G_IC_ (DCB)	2-layers EMAA mesh	[59]
				76	flexural modulus (ENF)		
	*CFs* + TGDDM-DETDA + EMAA	200 °C, 30 min, 20 kPa	217	55	G_IC_ (DCB)	2-step curing (5 h at 80 °C/8 h at 177 °C), 10 wt% EMAA pellets	[60]
		230 °C, 30 min, 20 kPa		105			
	*CFs* + DGEBA-DETDA + EMAA	150 °C, 30 min, 20 kPa	138	82	G_IC_ (DCB)	2-step curing (5 h at 80 °C/8 h at 177 °C), 10 wt% EMAA pellets	[61]
		200 °C, 30 min, 20 kPa		114			
	*CFs* + DGEBA-TETA + EMAA	US: 20 kHz, 1.1 kW (~150 °C)	142	135	G_IC_ (DCB)	4 EMAA meshes per ply interface	[62]
	*CFs* + DGEBA-TETA + PEGMA	150 °C, 30 min, 25 kPa	83	57	G_IC_ (DCB)	10 wt% thermopl. agent	[64]
	*CFs* + DGEBA-TETA + EMAA		97	156			
Thermoplastic melting and viscous flow into cracks upon heating	*CFs* + DGEBA-TETA + EVA	150 °C, 30 min, 25 kPa	97	103	G_IC_ (DCB)	10 wt% thermopl. agent	

* = value extrapolated/calculated from paper data or other papers with same formulation + = first healing cycle ^ = T_g_ of the epoxy (when formulation is a composite or includes a blend) italic = type of reinforcement.

**Table 3:** Summary of intrinsic self-healing formulations via chemical interactions.

Healing Mechanism	Formulation	Healing Conditions	T_g_	*η* ^+^	Test (Specimen)	Notes	Ref.
			(°C)	(%)			
Interdiffusion + DA/rDA	DGEBA-PACM + DGEBA-FA-BMI healing gel	10 µL gel on cracked surfaces, RT, 12 h, ~4.7 kPa	160 ^	37 ± 8	P_C_ (CT)	healing gel at 90 °C for 1 h prior to insertion	[79]
	DGEBA-FGE-PACM + BMI in DMF		56	70 ± 22	P_C_ (CT)	0.58 M of BMI in DMF	[80]
DA/rDA	2MF-DGEBA + BMI	150 °C, 5 min	42 ^	122	P_C_ (TDCB)	2-step synthesis, 20 wt% BMI	[83]
	*E-glass* + DGEBA-FGE-PACM + BMI in DMF	90 °C, 1 h and 22 °C, 12 h	~72 *	~55 *	Microdroplet single fiber pull-out test	*η* of fiber-resin interface, 0.25 w% FGE	[84]
	*CFs*-BMI + DGEBA-FGE-IPD	90 °C, 1 h and RT, 24 h	63	82	Microdroplet single fiber pull-out test	CFs oxidized w/HNO_3_, reacted w/TEPA, immerged into BMI (in DMF)	[86]
	*GR*-BMI + TF	800 W, 2 min (microwave), 4 min (IR)	100	~84 *	σ_T_ (dog-bone)	0,5 wt% graphene dispersed in NMP and BMI, TF added	[87]
	*GNS* + FDB-OGE-D230	~0.2 W cm^−2^, ~20 min (IR)	57	93	Lap shear strength	0.5 wt% GNS	[88]
	*MWCNTs* + EpF-BMI	140 °C, 40 min	71	~80	K_IC_	multiple healing cycles	[89]
	*B-GNPs* + DGEBA-FGE-BMI	130 °C, 2 h and 80 °C, 2 h	n.a.	~87	σ_F_ (dog-bone type V)	first study on bulk epoxy matrix healing	[90]
Alkoxyamines	diEP-DGEBA-DETA	90 °C, 1.5 h in Ar	~45 *	62	impact test	not feasible in air (O_2_ deactivation)	[91]
	diEP-DGEBA-PETMP	25 °C	0–10	60	σ_T_ (dog-bone)	sub-ambient T_g_	[92]
Imines	EN-VAN-AP	100 °C, 60 s, 10 N, then 120 °C, 4 h	71	~100 *	σ_T_ (dog-bone)	simple reprocessability	[93]
Tetrasulfides	EPS-4SH	65 °C, 20 min	−46	100	cohesion test, optical microscopy	cross-lining catalyzed w/1 wt% DMAP	[100]
	ER-OMAS	70 °C, 10 min, air, 30 kPa	−11	100	gap closure efficiency	~600 μm-thick film	[101]
		70 °C, 2 h		70	Interfacial strength recovery (SENT)	crack on healed SENT formed at same location as pristine SENT	
				60	K_IC_ (DENT)	J-integral for *η* evaluation	[102]
	*E-glass* + DGEBA-aliphatic amine-APTS-tetrathiol-TEA-BDS	85 °C, 16 h, 2 bar	~50 *	80 *	low-velocity impact (damaged area reduction)	composite prep by VARIM, impact energy 8 J	[103]
Transesterification	DGEBA-CBMI	200 °C, 2 h	125	~78	K_IC_ (SENT)	referred to highest CBMI wt%	[111]
	DGEBA-tricarballylic acid	150 °C, 18 h	56–59	59	σ_T_	compression programming	[112]
	DGEBA-phthalic anhydride	150 °C, 10 h, 12 MPa	98	88	σ_T_	powder state, 32 h milling time	[113]
Vitrimer (transesterification)	DGEBA-di/tricarboxylic acid	240 °C, 3 min	~15 *	~100 *	σ_T_ (dog-bone)	10 mol% Zn(acac)_2_ catalyst	[114]
	DGEBA-di/tricarboxylic acid	25% comp, 125 °C, 1 h	~15	~77 *	Lap shear P_C_	Zn(OAc)_2_ catalyst, Mohr clamp	[117]
	*MWCNTs* + DGEBA-adipic acid	~0.15 W cm^−2^, 30 s (IR)	~40	~80 *	P_C_ (film)	TBD catalyst, photo-welding	[119]
Vitrimer (aromatic disulfide exchange)	DGEBA-4AFD	200 °C, 5 min, 100 bar	130	~100	DMA measurements	mechanochromic effect	[122]
	BDSER-PPy	180 °C, 20 min, 20 MPa	133 ^	~100	σ_T_ (dog-bone)	reprocessable composites w/*MWCNTs*	[125]
Bio-Vitrimer (transesterification)	TEP-MHHPA	220 °C, 5 min	187	70	crack width reduction	Zn(acac)_2_ catalyst	[128]
	ESO-FPA	180 °C, 60 min	65	~100	optical microscopy		[129]
Bio-Vitrimer (disulfide exchange)	IS-ECH-4AFD	100 °C, 60 min	41	100	optical microscopy	multiple reprocessing cycles	[130]

* = value extrapolated/calculated from paper data or other papers with same formulation + = first healing cycle ^ = T_g_ of the epoxy (when formulation is a composite or includes a blend) *italic* = type of reinforcement.

## Data Availability

Data is contained within the article.

## Abbreviations

2-ME	2-mercaptoethanol
2MF	2-methyl furan
4AFD	4-aminophenyl disulfide
4SH	Pentaerythritol tetrakis(3-mercaptopropionate)
APTS	(3-Aminopropyl)trimethoxysilane
BDMA	benzyl dimethyl amine
BDS	Bis [3-(triethoxysilyl)propyl]disulfide
BDSER	bis-disulfide bond dynamic epoxy resin
B-GNP	bismaleimide-grafted graphite nanoplatelet
BMI	bismaleimide
CAI	compression after impact
CAN	covalent adaptable network
CBMI	chain-extended bismaleimide
CNF	carbon nanofiber
CT	compact tension
D230	polyether amine (commercial name)
DDS	4,4′-diaminodiphenylsulfone
DENT	double edge notched tension
DETA	diethylenetriamine
DGEBA	diglycidyl ether bisphenol A
DGEBF	diglycidyl ether bisphenol F
diEP	alkoxyamine-containing epoxy
DMA	dynamic mechanical analysis
DMAP	4-dimethylaminopyridine
DMF	dimethylformamide
DMTA	dynamic mechanical thermal analysis
DTDA	4,4′-dithiodianiline (4AFD synonym)
E	Young’s modulus
E_a_	activation energy
ECH	epichlorohydrin
EMA	poly(ethylene-co-methyl acrylate)
EMAA	poly(ethylene-co-methacrylic acid)
ENF	end-notched flexure
EN-VAN-AP	epoxy network w/vanillin and aminophenol
EpF	furan functionalized epoxy
EPS	epoxidized polysulfides (commercial name)
ER	epoxy resin
ESO	epoxy soybean oil
EVA	ethylene vinyl acetate
FA	furfurylamine
FDB	2,20-(Methylenebis(4,1-phenylene))bis(4-((oxiran-2-ylmethoxy)methyl)-3a,4,7,7a-tetrahydro-1H-4,7-epoxyisoin-dole-1,3(2H)-dione)
FGE	furan-epoxy monomer
FPA	fumaropimaric acid
FRP	fiber-reinforced polymer
FTIR	Fourier transform infrared spectroscopy
G_IC_	fracture energy (mode I)
GNS	graphite nanosheet
GR	graphene
HNO_3_	nitric acid
IPD	isophorone diamine
IR	infrared
IS	isosorbide
K_IC_	fracture toughness (Mode I)
MHHPA	4-methylcyclohexane-1,2-dicarboxylic anhydride
MWCNTs	multiwalled carbon nanotubes
NMA	nadic methylanhydride
NMP	N-Methyl-2-pyrrolidone
OGE	octanediol glycidyl ether
OMSA	organically modified silicone alkoxides
P	load
PACM	4,4′-methylene biscyclohexanamine
P_C_	load at failure/fracture
PCL	poly(ε-caprolactone)
PEGMA	poly (ethylene-co-glycidyl)-methacrylate
PETMP	pentaerythritoltetra(3-mercaptopropionate)
PMC	polymer matrix composite
PPy	polypyrrole
RT	room temperature
SEM	scanning electron microscopy/microscope
SENB	single-edge notched bending
SENT	single-edge notched tension
TBD	Triazabicyclodecene
TDCB	tapered double cantilever beam
TEA	triethylamine
TEP	triepoxy (vanillin + guaiacol + triphenol)
TEPA	tetraethylenepentamine
TETA	triethyltetramine
TF	tetra-furan monomer
T_g_	glass transition temperature
TGDDM	tetraglycidyl diamino diphenyl methane
TPB	three-point bending
U	strain energy
US	ultrasounds
VARIM	vacuum assisted resin infusion molding
WOF	work-of-fracture
Zn(acac)_2_	Zinc acetylacetonate hydrate
Zn(OAc)_2_	zinc acetate
Δl	displacement
*η*	healing efficiency
σ_X_	strength (T = tensile, C = compressive, I = impact, F = flexural)
